# Hydrastinine in Menorrhagia

**Published:** 1890-08

**Authors:** 


Hydrastinine in Menorrhagia.—Hydrastinine is obtained by oxydizing hydrastine. It may be given subcutaneously without pain, in doses of one grain of a five per cent. solution. Shortly after its administration there are light uterine pains. In ordinary cases of menorrhagia it acts well. In cases of pyosalpinx it has acted favorably. The usual formula is as follows :

| | |
|---|---|
| ℞—Hydrastinine (P., D. & Co.'s) | gr. i. |
| Aquæ dest. | ʒ ii. |

M. Sig.—Six minims once or twice daily.

In hemorrhage due to endometritis or myomata, the percentage of favorable results has been large. No evidence of the effect of hydrastinine in labor is furnished, but it is probable that it will be favorable, because it contracts the vessels, and uterine pains result. The best time for its use is six to eight days before menstruation, which was rendered profuse by textural changes in the uterus.—*Therap. Monatschrifte.*

